# Supplementary material for: Effects of Region, Processing, and Their Interaction on the Elemental Profiles of Pu-Erh Tea
Source: Foods. 2025 Aug 17;14(16):2848. doi: 10.3390/foods14162848 (PMC12385689; doi:10.3390/foods14162848)
Supplement: Supplementary file 1 [file foods-14-02848-s001.zip › foods-3775875-supplementary.pdf]

Table S1. Temperature and moisture content recorded during different turning stages of pile-fermentation.

**Region**	**Turning**	**Time**	**Temperature (℃)**	**Moisture content(%)**
Jinggu	First turning	2023.6.8-2023.6.15	46	43
	Second turning	2023.6.15-2023.6.23	48	51
	Third turning	2023.6.23-2023.7.1	53	52
	Fourth turning	2023.7.1-2023.7.7	58	54
Bangdong	First turning	2023.6.8-2023.6.15	53	56
	Second turning	2023.6.15-2023.6.23	54	36
	Third turning	2023.6.23-2023.7.1	50	38
	Fourth turning	2023.7.1-2023.7.7	48	35
Ning′er	First turning	2023.6.8-2023.6.15	43	46
	Second turning	2023.6.15-2023.6.23	47	53
	Third turning	2023.6.23-2023.7.1	56	54
	Fourth turning	2023.7.1-2023.7.7	59	53

Table S2. Quality control for determining the elements of tea.

Element	Isotope	LOD (μg/kg)	LOQ (μg/kg)	Certified value(mg/kg)	Exptl value(mg/kg)	Recovery(%)
Li	7	0.007	0.023	0.25±0.02	0.26±0.00	104±0
K	39	5.466	18.220	14500±500	17201±159	119±1
Ca	43	5.565	18.550	4700±200	4561±78	97±2
Sc	45	0.008	0.027	0.089±0.017	0.089±0.003	100±3
Fe	56	0.132	0.440	149±7	148±4	99±3
Co	59	0.003	0.010	0.28±0.002	0.25±0.005	89±2
Cu	63	0.005	0.017	8.3±0.5	7.4±0.1	89±1
Zn	66	0.175	0.583	27±3	27±0	100±0
As	75	0.031	0.103	0.10±0.02	0.11±0.00	110±0
Rb	85	0.189	0.630	45.9±3.3	54.6±0.3	119±0
Sr	88	0.025	0.083	13.7±0.7	15.0±0.1	109±1
Y	89	0.003	0.010	2.0±0.2	2.2±0.0	110±0
Cd	111	0.001	0.003	0.046±0.005	0.052±0.001	113±2
Cs	133	0.008	0.027	0.19±0.02	0.20±0.01	105±5
Ba	137	0.07	0.233	32±3	37±0	115±1
La	139	0.002	0.007	1.01±0.08	1.2±0.02	119±2
Nd	146	0.003	0.010	0.66±0.08	0.75±0.00	114±0
Sm	147	0.001	0.003	0.120±0.020	0.135±0.003	113±3
Eu	153	0.001	0.003	0.027	0.029±0.001	107±4
Gd	157	0.001	0.003	0.140±0.020	0.147±0.002	105±1
Tb	159	0.001	0.003	0.021±0.002	0.023±0.000	110±0
Dy	163	0.002	0.007	0.130±0.020	0.146±0.001	110±1
Ho	165	0.001	0.003	0.028±0.004	0.031±0.000	112±0
Er	166	0.001	0.003	0.080±0.010	0.095±0.000	119±0
Tm	169	0.001	0.003	0.014±0.002	0.015±0.000	107±0
Yb	172	0.001	0.003	0.100±0.020	0.105±0.001	105±1
Lu	175	0.001	0.003	0.016±0.003	0.017±0.000	106±0
Pb	206	0.017	0.057	1.09±0.13	1.21±0.02	111±2

Table S3. Mineral contents (mean ± SD) in Pu-erh tea from three different regions at various processing stages.

Element	Region	Fresh leaves	Kill-green	Rolling	First turning	Second turning	Third turning	Fourth turning	Ripe Pu-erh tea
Li (ug/kg)	Jinggu*	58.66±16.11^{ab}	57.20±2.60^{ab}	58.88±1.36^{a}	65.82±3.94^{ab}	64.28±2.23^{ab}	64.37±0.74^{ab}	66.99±1.14^{b}	78.15±4.91^{ab}
	Bangdong	88.75±15.12	58.75±0.81	57.08±4.60	59.56±2.78	70.26±7.33	70.76±5.49	82.99±7.03	74.48±2.89
	Ning'er**	15.86±0.93^{d}	33.58±2.21^{c}	35.40±1.72^{bcd}	61.07±1.07^{ab}	53.91±4.41^{abcd}	54.88±1.96^{a}	51.80±3.41^{abc}	67.22±2.20^{ab}
K (mg/kg)	Jinggu	25308±1411	26335±323	26792±580	25613±378	25638±387	25617±44	26201±581	28477±1103
	Bangdong**	24884±517^{cd}	22310±164^{bc}	22080±214^{bc}	26670±589^{abcd}	31463±1110^{abcd}	30178±1033^{abcd}	33407±564^{ad}	33104±476^{a}
	Ning'er*	21640±516^{a}	22484±416^{a}	22005±378^{ab}	21504±287^{ab}	22549±295^{ab}	22498±259^{ab}	21609±2635^{ab}	25240±470^{b}
Ca (mg/kg)	Jinggu	3760±310	4060±180	4030±110	3500±90	3850±60	3710±60	3990±50	4570±220
	Bangdong**	5310±40^{a}	3400±30^{b}	3470±70^{b}	4210±130^{ab}	4930±180^{ab}	4620±190^{ab}	4820±200^{ab}	5500±130^{a}
	Ning'er*	3900±210^{abcd}	3890±100^{ab}	3760±100^{abcd}	2920±50^{abcd}	3210±110^{cd}	3160±40^{ac}	3260±310^{abcd}	3460±20^{bd}
Sc (ug/kg)	Jinggu	38.407±9.390	45.244±1.716	51.756±2.701	46.775±2.343	49.346±2.299	43.732±1.770	46.112±2.391	48.600±3.349
	Bangdong	33.507±3.704	29.732±1.780	23.724±1.810	26.855±2.132	33.913±1.054	34.582±2.311	39.744±3.604	33.501±4.357
	Ning'er	7.825±1.488	11.793±0.917	11.575±0.782	13.478±0.653	14.967±1.635	15.235±3.567	15.203±0.671	16.889±1.675
Fe (mg/kg)	Jinggu*	86±27^{ab}	92±3^{a}	98±4^{ab}	114±1^{ab}	118±5^{ab}	117±2^{b}	132±8^{ab}	141±10^{ab}
	Bangdong**	120±1^{abc}	113±9^{a}	103±3^{ab}	134±3^{abc}	158±7^{abc}	154±13^{abc}	194±8^{c}	168±8^{bc}
	Ning'er**	53±1^{a}	66±2^{a}	71±2^{ab}	83±5^{ab}	83±0^{b}	86±8^{ab}	81±8^{ab}	100±1^{c}
Co (ug/kg)	Jinggu**	136.57±80.39^{ab}	124.56±5.21^{ab}	110.99±2.33^{b}	136.17±1.58^{a}	140.11±1.39^{a}	137.34±2.83^{a}	139.29±1.88^{a}	147.42±6.81^{ab}
	Bangdong**	135.11±3.77^{a}	116.38±1.56^{a}	112.89±2.42^{a}	255.61±11.05^{ab}	280.21±2.68^{b}	272.16±6.96^{b}	305.70±12.81^{b}	286.94±10.22^{b}
	Ning'er**	37.51±0.92^{a}	52.05±2.45^{abc}	54.12±2.15^{abc}	58.55±0.81^{abc}	60.87±1.27^{b}	65.24±5.00^{abc}	64.39±4.58^{abc}	71.59±0.57^{c}
Cu (mg/kg)	Jinggu**	14.1±2.5^{ab}	15.8±0.1^{b}	16.5±0.4^{ab}	17.2±0.2^{ab}	16.8±0.1^{a}	17.2±0.1^{a}	17.2±0.4^{ab}	18.7±0.8^{ab}
	Bangdong**	12.1±0.1^{a}	7.4±0.1^{b}	8.9±0.2^{c}	14.6±0.3^{abcd}	17.1±0.4^{d}	16.1±0.4^{d}	17.5±0.2^{d}	17.7±0.2^{d}
	Ning'er**	17.7±0.2^{a}	15.1±0.3^{b}	15.9±0.3^{ab}	16.5±0.4^{ab}	16.3±0.1^{b}	16.7±0.2^{a}	16.2±1.3^{ab}	17.7±0.3^{a}
Zn (mg/kg)	Jinggu**	35±7^{ab}	32±0^{ab}	31±0^{b}	33±0^{ab}	34±0^{a}	34±0^{a}	34±1^{av}	36±2^{ab}
	Bangdong**	27±0^{a}	27±0^{a}	27±0^{a}	34±0^{ab}	40±2^{ab}	37±2^{ab}	42±1^{b}	40±1^{b}
	Ning'er*	37±1^{ab}	37±1^{ab}	37±1^{ab}	38±1^{ab}	37±0^{a}	38±0^{ab}	37±4^{ab}	41±0^{b}
As	Jinggu	50.02±17.63	50.36±5.26	48.47±3.28	57.71±6.07	54.34±4.72	53.41±2.71	60.37±4.11	64.67±11.62

(ug/kg)	Bangdong	53.84±2.37	44.52±2.67	47.22±11.73	47.78±0.84	53.37±0.92	49.92±1.45	61.33±8.26	63.71±9.65
	Ning′er*	20.03±5.05^{ab}	26.02±1.60^{a}	32.32±2.80^{ab}	43.20±0.35^{ab}	43.99±0.78^{b}	51.48±7.80^{ab}	47.40±7.43^{ab}	55.04±7.48^{ab}
Rb (mg/kg)	Jinggu*	96.8.±26.1^{abcd}	120.3±2.0^{ab}	121.1±3.1^{abcd}	111.0±1.1^{ac}	103.2±0.5^{abcd}	104.3±0.8^{bd}	105.1±1.1^{cd}	110.0±5.1^{abcd}
	Bangdong**	117.1±1.8^{ab}	109.3±1.2^{ab}	104.0±2.6^{a}	131.1±1.7^{abc}	155.2±5.1^{bc}	148.5±4.0^{c}	164.6±2.7^{c}	161.3±2.4^{c}
	Ning′er**	35.6±0.2^{ac}	37.4±0.6^{a}	38.2±0.4^{abcd}	39.3±0.3^{abcd}	41.3±0.7^{bc}	40.9±0.6^{abc}	40.5±4.7^{abcd}	46.7±0.4^{d}
Sr (mg/kg)	Jinggu*	11.1±5.3^{ab}	16.9±1.1^{ab}	15.1±1.1^{ab}	10.0±0.3^{a}	11.0±0.1^{ab}	10.6±0.2^{ab}	12.2±0.3^{b}	13.5±0.7^{ab}
	Bangdong**	16.5±0.3^{b}	9.5±0.1^{a}	9.8±0.2^{a}	12.1±0.2^{abc}	13.9±.30^{c}	13.5±0.0^{bc}	14.1±0.5^{abc}	14.8±0.3^{bc}
	Ning′er**	7.8±0.3^{ad}	4.3±0.1^{bd}	4.2±0.0^{abcd}	4.6±0.1^{abcd}	5.2±0.2^{cd}	5.0±0.1^{abc}	5.5±0.5^{abcd}	5.8±0.1^{d}
Y (ug/kg)	Jinggu*	215.2±72.2^{abcd}	268.2±3.5^{ab}	303.0±9.2^{ac}	247.8±4.6^{abcd}	271.2±21.2^{abcd}	241.5±2.9^{cd}	241.3±7.0^{bd}	268.7±14.3^{abcd}
	Bangdong**	196.5±1.1^{a}	86.4±7.6^{d}	73.3±3.4^{d}	178.9±11.9^{abcd}	193.6±8.1^{ac}	202.9±5.6^{ac}	220.5±1.5^{bc}	219.4±4.2^{ac}
	Ning′er*	32.0±3.8^{a}	60.1±3.0^{ab}	61.3±3.9^{b}	60.6±1.8^{ab}	61.6±1.7^{ab}	63.0±1.8^{ab}	76.7±21.2^{ab}	71.7±2.0^{ab}
Cd (ug/kg)	Jinggu*	27.748±10.054^{ab}	36.000±1.763^{b}	37.062±2.019^{ab}	47.782±1.076^{a}	47.522±0.548^{ab}	42.857±1.214^{ab}	45.421±1.386^{ab}	50.865±2.508^{a}
	Bangdong**	23.670±1.167^{a}	14.140±0.370^{b}	15.630±1.152^{b}	50.674±1.779^{abc}	54.130±6.037^{abc}	51.689±4.186^{abc}	60.247±0.948^{c}	56.816±1.500^{c}
	Ning′er*	26.676±0.782^{a}	44.377±1.949^{b}	42.968±1.783^{ab}	22.550±0.616^{ab}	28.765±0.857^{a}	26.226±1.141^{a}	29.933±2.679^{ab}	31.560±1.061^{ab}
Cs (ug/kg)	Jinggu	241.05±164.58	277.14±4.70	266.58±3.51	271.49±6.74	241.45±1.68	253.79±4.91	253.42±2.63	261.61±12.76
	Bangdong**	211.03±7.57^{b}	497.78±15.84^{d}	468.14±9.80^{cd}	227.03±6.24^{abc}	263.75±3.45^{ab}	255.17±10.49^{ab}	286.66±7.66^{a}	278.55±4.81^{a}
	Ning′er**	129.88±2.65^{d}	158.20±6.64^{abcd}	164.69±0.74^{abcd}	167.41±5.51^{abcd}	160.93±3.63^{a}	168.53±1.35^{ab}	158.28±18.69^{abcd}	184.52±2.46^{bc}
Ba (mg/kg)	Jinggu*	17±6^{ab}	24±1^{a}	22±2^{ab}	15±0^{b}	16±0^{ab}	16±0^{ab}	19±2^{ab}	19±1^{ab}
	Bangdong**	44±1^{a}	12±0^{bd}	13±0^{c}	33±1^{abcd}	37±1^{ad}	36±2^{abcd}	38±0^{a}	39±1^{a}
	Ning′er**	23±0^{a}	18±1^{b}	19±0^{avc}	12±0^{abc}	12±0^{c}	13±0^{bc}	13±1^{bc}	13±0^{bc}
La (ug/kg)	Jinggu	198.63±108.97	161.34±1.92	169.78±9.67	189.30±22.26	188.34±8.26	179.18±14.74	176.81±9.38	193.27±8.04
	Bangdong**	237.73±17.27^{a}	111.00±6.67^{a}	77.84±4.54^{a}	186.77±14.47^{ab}	219.14±22.22^{ab}	199.57±8.63^{b}	229.02±10.10^{b}	225.11±5.16^{b}

		b							
	Ning'er**	26.30±1.18^{d}	42.42±1.28^{a}	45.06±3.33^{abcd}	48.07±2.39^{abcd}	54.41±4.74^{abcd}	48.41±0.41^{ac}	72.57±40.16^{abcd}	61.22±2.52^{bc}
Nd (ug/kg)	Jinggu	192.35±81.39	175.87±1.57	196.07±17.03	180.44±3.22	197.74±22.74	173.42±16.06	174.50±9.46	188.48±4.55
	Bangdong**	151.38±15.73^{ab}	92.25±9.96^{ab}	57.69±2.21^{a}	129.07±16.79^{ab}	145.20±17.38^{ab}	137.50±5.86^{b}	153.87±1.48^{b}	150.64±4.57^{b}
	Ning'er*	22.58±1.73^{a}	36.90±2.44^{b}	39.51±3.52^{ab}	43.44±2.24^{ab}	52.87±6.73^{ab}	43.76±3.43^{ab}	55.43±23.08^{ab}	51.25±0.43^{b}
Sm (ug/kg)	Jinggu	42.486±16.169	39.306±0.660	46.083±1.391	40.475±3.158	43.634±1.934	39.956±3.880	40.634±2.328	42.641±3.859
	Bangdong**	30.263±2.982^{ab}	17.421±0.214^{ab}	10.318±1.241^{a}	24.511±0.882^{ab}	27.691±5.191^{ab}	25.885±1.080^{b}	29.553±2.331^{b}	26.959±1.841^{b}
	Ning'er	4.385±0.315	9.932±1.186	8.029±0.453	9.198±0.504	10.236±2.015	9.245±0.687	11.781±4.300	11.534±1.406
Eu (ug/kg)	Jinggu	9.480±2.688	10.643±0.438	12.771±1.111	10.051±0.593	10.640±0.529	9.744±0.395	9.983±0.554	10.732±0.630
	Bangdong**	6.803±0.489^{a}	2.842±0.179^{c}	2.450±0.351^{c}	5.309±0.827^{b}	6.544±0.294^{a}	6.409±0.598^{a}	6.743±0.511^{a}	7.389±1.047^{a}
	Ning'er	1.867±0.141	2.562±0.281	2.842±0.292	2.638±0.252	2.734±0.050	2.388±0.317	2.692±0.981	2.478±0.298
Gd (ug/kg)	Jinggu	41.480±11.465	41.725±1.008	50.404±3.304	41.317±1.855	43.064±2.390	40.927±1.345	40.896±1.389	44.775±2.725
	Bangdong**	29.862±1.960^{ad}	14.381±1.533^{ab}	10.938±0.130^{bc}	24.260±3.118^{abcd}	27.014±1.831^{d}	27.636±2.034^{ad}	30.258±2.035^{ad}	31.805±1.304^{abcd}
	Ning'er	4.903±0.750	10.718±1.391	9.935±0.791	10.639±0.809	11.757±1.277	11.037±1.091	11.809±3.072	12.718±0.012
Tb (ug/kg)	Jinggu	6.064±2.013	7.122±0.366	7.965±0.808	6.528±0.403	6.502±0.099	6.424±0.420	6.050±0.104	6.569±0.520
	Bangdong**	4.340±0.252^{ab}	2.224±0.077^{bc}	1.657±0.128^{c}	3.555±0.075^{abc}	3.868±0.402^{abc}	4.079±0.583^{abc}	4.760±0.107^{a}	4.304±0.199^{a}
	Ning'er*	0.770±0.137^{a}	1.426±0.061^{ab}	1.488±0.042^{ab}	1.511±0.020^{ab}	1.666±0.194^{ab}	1.738±0.424^{ab}	1.788±0.376^{ab}	1.778±0.115^{b}
Dy (ug/kg)	Jinggu*	35.078±9.809^{abcd}	43.841±1.107^{ab}	50.942±2.266^{ac}	37.230±0.952^{cd}	40.640±1.500^{abcd}	37.684±2.002^{bd}	39.085±2.328^{abcd}	40.342±3.705^{abcd}
	Bangdong**	26.707±1.047^{b}	12.566±0.853^{a}	9.958±1.504^{a}	22.045±2.000^{ab}	25.412±1.178^{b}	24.737±2.917^{ab}	29.015±1.436^{b}	26.700±0.469^{b}
	Ning'er**	4.119±0.348^{a}	8.771±0.740^{ab}	8.506±0.858^{ab}	7.572±0.713^{ab}	8.698±0.528^{b}	8.654±0.383^{b}	9.268±1.145^{ab}	10.206±0.496^{b}
Ho (ug/kg)	Jinggu**	7.300±1.694^{c}	9.149±0.519^{ab}	10.059±0.740^{a}	7.800±0.294^{bc}	8.495±0.263^{bc}	7.917±0.416^{bc}	7.949±0.532^{bc}	8.476±0.202^{bc}
	Bangdong**	5.639±0.189^{a}	2.587±0.202^{bc}	2.047±0.214^{b}	4.689±0.328^{abc}	5.358±0.318^{a}	5.218±0.397^{ac}	5.752±0.168^{a}	5.885±0.289^{a}

	Ning′er*	0.730±0.173^{a}	1.799±0.097^{ab}	1.778±0.076^{ab}	1.809±0.196^{ab}	1.617±0.092^{ab}	1.687±0.131^{ab}	1.947±0.432^{ab}	2.130±0.190^{b}
Er (ug/kg)	Jinggu*	20.202±5.733^{c}	26.995±2.244^{ab}	28.536±2.207^{a}	23.669±1.385^{abc}	23.460±1.774^{bc}	23.825±0.634^{abc}	24.140±1.432^{abc}	25.253±1.131^{ab}
	Bangdong**	15.785±0.491^{c}	7.518±0.309^{ab}	5.705±0.450^{a}	14.466±0.590^{abcd}	15.055±0.946^{b}	15.332±0.710^{bc}	17.935±1.038^{bcd}	17.345±1.151^{bcd}
	Ning′er**	2.238±0.103^{a}	4.599±0.539^{ab}	4.387±0.113^{ab}	4.277±0.278^{ab}	4.823±0.592^{ab}	4.579±0.070^{b}	5.190±0.966^{ab}	5.595±0.297^{b}
Tm (ug/kg)	Jinggu	3.034±0.768	4.176±0.428	4.514±0.439	3.747±0.285	3.639±0.119	3.505±0.062	3.531±0.217	3.533±0.081
	Bangdong**	2.421±0.402^{ab}	1.052±0.176^{a}	0.851±0.101^{a}	2.084±0.056^{ab}	2.037±0.261^{b}	2.249±0.151^{b}	2.766±0.127^{b}	2.570±0.177^{b}
	Ning′er	0.340±0.063	0.587±0.055	0.522±0.029	0.715±0.033	0.668±0.056	0.624±0.060	0.731±0.119	0.698±0.042
Yb (ug/kg)	Jinggu*	18.568±3.028^{ab}	27.761±0.318^{a}	29.872±2.489^{ab}	25.090±0.300^{b}	24.937±0.670^{ab}	24.748±0.619^{ab}	23.622±0.771^{ab}	24.558±0.599^{ab}
	Bangdong**	13.937±0.604^{b}	6.928±0.606^{a}	5.188±0.555^{a}	13.054±0.561^{ab}	14.636±0.884^{b}	13.605±1.645^{ab}	16.597±0.367^{b}	16.130±0.468^{b}
	Ning′er*	2.182±0.365^{a}	3.546±0.207^{ab}	3.793±0.243^{ab}	3.598±0.409^{ab}	3.669±0.370^{ab}	3.722±0.217^{ab}	3.727±0.672^{ab}	4.629±0.410^{b}
Lu (ug/kg)	Jinggu**	2.944±0.826^{d}	4.646±0.460^{a}	4.422±0.280^{ab}	3.694±0.196^{bcd}	3.777±0.362^{bc}	3.452±0.185^{cd}	3.702±0.234^{bcd}	3.636±0.221^{cd}
	Bangdong**	2.064±0.165^{b}	1.123±0.258^{ab}	0.735±0.104^{a}	1.885±0.021^{ab}	1.795±0.313^{ab}	2.271±0.112^{b}	2.305±0.089^{b}	2.383±0.191^{b}
	Ning′er	0.277±0.124	0.521±0.081	0.514±0.050	0.572±0.098	0.550±0.102	0.537±0.083	0.624±0.111	0.661±0.074
Pb (ug/kg)	Jinggu	109.001±27.710	150.743±32.890	145.766±13.924	154.608±13.071	158.103±8.799	167.133±4.082	2045.518±3187.927	199.107±16.286
	Bangdong	152.043±2.313	140.840±13.681	166.239±26.123	155.313±17.837	182.928±39.110	184.891±13.109	220.3987±36.451	247.467±98.361
	Ning′er	88.442±6.092	94.543±3.648	130.860±26.463	177.210±62.182	148.375±39.897	137.178±25.359	140.559±14.086	191.621±41.633

Data are shown as the mean ± standard deviation. $^{a–c}$ in the same row indicate that there are significant differences among regions at $p < 0.05$ level, as determined by one-way ANOVA followed by Duncan's multiple range test. * means significant difference ($p < 0.05$), ** means highly significant difference ($p < 0.01$).

Table S4. The percentages of total square variance of each effect on each element.

Source of Variation	Region (R)	Degree of freedom	Processing process (P)	Degree of freedom	R×P	Degree of freedom	Error
Li (ug/kg)	3612.18**	2	612.77**	7	396.28**	14	31.84
K (mg/kg)	194540146**	2	33031051**	7	19597648**	14	658132
Ca (mg/kg)	71336**	2	9047**	7	8262**	14	223
Sc (ug/kg)	6521.455**	2	56.661**	7	46.184**	14	8.964
Fe (mg/kg)	25541**	2	3619**	7	452**	14	63
Co (ug/kg)	158826.61**	2	10169.59**	7	5735.76**	14	295.27
Cu (mg/kg)	56.87**	2	29.29**	7	13.46**	14	0.43
Zn (mg/kg)	97**	2	62**	7	34**	14	3
As (ug/kg)	1569.87**	2	496.33**	7	103*	14	45
Rb (mg/kg)	59202**	2	578**	7	712**	14	34
Sr (mg/kg)	451.3**	2	8.1**	7	15.7**	14	1.4
Y (ug/kg)	232283.9**	2	2775.8**	7	5005.7**	14	286.9
Cd (ug/kg)	768.285**	2	496.019**	7	506.903**	14	8.368
Cs (ug/kg)	137909.43**	2	14834.66**	7	11118.99**	14	1185.97
Ba (mg/kg)	1739**	2	97**	7	215**	14	2
La (ug/kg)	143987.76**	2	5406.59**	7	3098.20**	14	659.86
Nd (ug/kg)	121763.25**	2	1455.13*	7	1376.27**	14	392.55
Sm (ug/kg)	6398.761**	2	45.023**	7	63.293**	14	16.168
Eu (ug/kg)	389.423**	2	1.968**	7	6.103**	14	0.601
Gd (ug/kg)	6429.926**	2	49.412**	7	89.163**	14	8.708
Tb (ug/kg)	159.976**	2	0.510	7	2.292**	14	0.271
Dy (ug/kg)	6332.469**	2	24.759**	7	103.271**	14	6.377
Ho (ug/kg)	271.040**	2	1.137**	7	4.174**	14	0.219
Er (ug/kg)	2417.417**	2	14.572**	7	34.209**	14	2.418
Tm (ug/kg)	57.825**	2	0.216**	7	0.949**	14	0.062
Yb (ug/kg)	2742.952**	2	12.306**	7	36.830**	14	0.989
Lu (ug/kg)	64.37**	2	0.22**	7	0.87**	14	0.07
Pb (ug/kg)	10928.397**	2	8483.085**	7	1011.182	14	1064.228

Asterisk(*)means significant difference (p <0.05),asterisk(**)means highly significant difference (p <0.01).

Table S5. Principal component analysis table of characteristic mineral elements.

Element	Component		
	1	2	3
Li (ug/kg)	4.614	2.435	-1.125
K (mg/kg)	5.161	4.234	0.479
Ca (mg/kg)	4.148	4.224	-0.485
Sc (ug/kg)	6.447	-1.836	-0.381
Fe (mg/kg)	4.818	4.539	-0.777
Co (ug/kg)	4.612	4.758	-0.732
Cu (mg/kg)	1.703	1.385	4.811
Zn (mg/kg)	-0.406	4.225	3.850
As (ug/kg)	4.702	1.424	-0.054
Rb (mg/kg)	5.681	2.684	-1.898
Sr (mg/kg)	5.675	0.911	-1.734
Y (ug/kg)	6.650	-1.198	0.353
Cd (ug/kg)	4.110	3.369	2.434
Cs (ug/kg)	2.026	-0.079	-4.198
Ba (mg/kg)	3.244	4.769	-0.706
La (ug/kg)	6.244	1.595	-0.641
Nd (ug/kg)	6.557	-1.119	-0.040
Sm (ug/kg)	6.367	-2.136	0.333
Eu (ug/kg)	6.308	-2.318	0.726
Gd (ug/kg)	6.460	-2.097	0.577
Tb (ug/kg)	6.359	-2.409	0.562
Dy (ug/kg)	6.414	-2.309	0.486
Ho (ug/kg)	6.438	-2.228	0.444
Er (ug/kg)	6.478	-2.069	0.442
Tm (ug/kg)	6.380	-2.268	0.362
Yb (ug/kg)	6.365	-2.405	0.474
Lu (ug/kg)	6.251	-2.589	0.459
Pb (ug/kg)	3.072	3.722	0.071
Variance contribution rate (%)	62.261	14.927	9.567
Cumulative variance contribution rate (%)	62.261	77.189	86.757
